# Childhood Separation From Parents and Self-Harm in Adolescence: A Cross-Sectional Study in Mainland China

**DOI:** 10.3389/fpsyg.2021.645552

**Published:** 2022-01-26

**Authors:** Tao-Jie Zhou, Meng-Yuan Yuan, Hao-Yang Ren, Guo-Die Xie, Geng-Fu Wang, Pu-Yu Su

**Affiliations:** ^1^Department of Clinical Medicine, The Second School of Clinical Medicine, Anhui Medical University, Hefei, China; ^2^Department of Maternal, Child and Adolescent Health, School of Public Health, Anhui Medical University, Hefei, China; ^3^Anhui Provincial Key Laboratory of Population Health and Aristogenics, Hefei, China; ^4^Key Laboratory of Population Health Across Life Cycle, Ministry of Education of the People’s Republic of China, Anhui Medical University, Hefei, China; ^5^NHC Key Laboratory of Study on Abnormal Gametes and Reproductive Tract, Hefei, China

**Keywords:** self-harm, parent-child separation, left-behind children, adolescents, adversities

## Abstract

As the prevalence of self-harm among adolescents in Chinese escalates, finding out the potential risk factors associated with self-harm behaviors has aroused much attention. This study aims to explore the association between parent-child separation and series of self-harm (SH) subtypes among Chinese adolescents. We survey a total of 4,928 middle school students aged from 12 to 18 years at school. Parent-child separation was investigated from four dimensions—occurrence of parental separation, separation status, age at first separation and duration of separation. Self-harm series are deemed as five subtypes—highly lethal self-harm, less lethal self-harm with visible tissue damage, self-harm without visible tissue damage, self-harmful behaviors with latent damage and psychological self-harm. Multivariate logistic regression is used to explore the associations between parent-child separation and different subtypes of self-harm among adolescents. Paternal separation is associated with each type of self-harm whilst maternal separation is not correlated with highly lethal self-harm. Except for highly lethal self-harm, the other four subtypes of self-harm demonstrate a relation with both length of paternal separation and maternal separation with aOR ranging from 1.02 to 1.06. Individuals who suffer parental separation prior to the age of three were at a higher risk for four types of less-lethal self-harm. The association of parent-child separation with self-harm deserves our attention, and future research is needed to identify the underlying mechanisms.

## Introduction

Over the past three decades, inspired by the policy of reform and opening-up and the development of economy in China, more and more rural laborers choose to move into urban areas to make a living (Xu et al., 2018), which is considered the largest migration in history (Wen and Lin, 2012). Working-age parents are forced to migrate, leaving their young children behind largely because of primarily financial pressure and lack of access to public services (Valtolina and Colombo, 2012). When parents leave their children at home and move into cities to look for better employment opportunities, their children who are left at home living with grandparents are referred to as “left-behind children” (LBC) (Jia and Tian, 2010). There are large numbers of left-behind children separated from their parents in the Chinese society. Migration from rural to urban areas has driven the number of left-behind children up to 61 million, accounting for nearly 25% of adolescents in China (Yuan and Wang, 2016). Hence, parent-child separation has become increasingly common in China during the past decades.

Self-harm has become a major social problem among adolescents. Generally, it can be divided into two separate groups: non-suicidal self-injury (NSSI) and suicide attempts (Hawton et al., 2012). Previous studies have reported that adolescents were more likely to be involved in NSSI and revealed an increasing trend of NSSI among adolescents (Hawton et al., 2002; Yang and Feldman, 2017). In addition, NSSI was considered as a factor of high risk for suicidal behaviors (Cox et al., 2012; Ribeiro et al., 2016). The reasons for increasing prevalence of SH in adolescents may be ascribed to individual, familial, as well as environmental factors (Tian et al., 2019).

Due to the absence of consensus in the definition of SH, the prevalence reported varies significantly in diverse studies. The prevalence of NSSI distinctly ranges from 6.4 to 47.5% in China (Lang and Yao, 2018). A meta-analysis suggests that the pooled prevalence of NSSI ranges from 5.5 to 17.2% among adolescents after adjusting for methodological factors (Swannell et al., 2014). Considering that the terms for NSSI varies considerably in different countries (Skegg, 2005), it is hard to draw a comparison of self-harm prevalence among adolescents.

However, dichotomous classification of self-harm, known as NSSI and suicide attempts, only focus on the obvious damage to individuals, whereas neglecting the implicit forms of self-harm. There is still a lack of studies which have taken psychological self-harm into consideration. Besides, it is hard to distinguish self-harm from suicidal attempts (Hawton et al., 2012). Therefore, we consider one type of classification in terms of the severity of the negative impact of SH (Han et al., 2018): highly lethal self-harm, less lethal self-harm with visible tissue damage, self-harm without visible tissue damage, self-harmful behaviors with latent damage, and psychological self-harm. These five types would provide a better understanding of self-harm.

Object relations theories offer an explanation of both how the experience of the child-parent separation relates to self-harm and how self-harm develops as a result of people’s early psychological history (Hamilton, 1989). Furthermore, attachment theories also emphasize that less contact with their parents would have bad effect on later development in adolescents (Anglin et al., 2008). Exposure to parent-child separation was associated with a host of negative physical and mental health outcomes. It is reported that adolescents who have previously experienced separation were more likely to suffer from loneliness, anxiety, self-esteem and depression (Liu et al., 2009; Jia and Tian, 2010; Luo et al., 2012). A meta-analysis also suggest that parental separation is harmful for adolescents’ health, showing left-behind children are more susceptible to depression, anxiety, suicidal ideation, conduct disorder, substance use (Fellmeth et al., 2018). Consistently, a prospective study finds that separation occurrence, age of separation, and duration of separation are associated with both schizophrenia and bipolar disorder (Paksarian et al., 2015). Moreover, a cross-sectional survey has illustrated that LBC living with one parent have better psychological health than those living with grandparents (Jia and Tian, 2010). Additionally, children who were left at home by their parents during childhood may alienate their parents, even though the original purpose of parents was to give them a better life (Zhao et al., 2018). During World War II those who experienced evacuation unaccompanied by either parent tend to suffer from the long-term outcome of more severely depressive symptoms compared to those without any parental separation (Pesonen et al., 2007). Specifically, the relationship of parental absence and unhealthy behaviors among adolescents in rural China is well documented (Gao et al., 2010). Thus, considering the adverse effects of parent-child separation and the prevalence of left-behind children in Chinese background, it is urgent to unveil the possible correlation between parental separation and later development.

There is growing concern with increased risk for self-injuries as a consequence of parental separation around the world. A previous study indicates a connection between poor parent-child attachment and suicidal behavior (Fergusson et al., 2000). Another research reveals that parental separation is the risk factor for suicide attempts and a sense of being neglected will ultimately lead to NSSI (Guvendeger Doksat et al., 2017). Moreover, the experience of being left-behind increases the likelihood of suicide risk promoted by life stresses, depression, mental disorder, and lack of social support (Zhou et al., 2019). A recent study suggests all the three types parental separation—paternal separation, maternal separation, and both-parents separation are significantly correlated with negative emotions like suicide ideation (Fu et al., 2017).

However, to date, little evidence exists of any effect of parental separation on five subtypes of self-harm among Chinese adolescents. Hence, we hypothesized that experiences of parental separation would be a risk factor to adolescents’ self-harm behavior in the general population regardless of gender. In order to precisely estimate the effect of parent-child separation on potential self-harm, we examine the association between parental leave and SH in adolescents by conducting a cross-sectional study in China involving 4,928 general students. The aims of present study are to explore possible relationships between characteristics of parent-child separation (i.e., separation occurrence, age at first separation, separation status, and duration of parental separation) and the five SH subtypes. The definition of self-harm is regarded as a series of intentions and behaviors, which may help shed light on the essential period and provide novel insight into comprehensive understanding of self-harm.

Using data collected from a large case–sectional study in mainland China, we aimed to figure out the following questions: (1) are individuals who experienced parental separation in childhood differ from those who do not concerning demographic features?; (2) is parental separation associated with an increased risk of self-harm in adolescent?; (3) are separation status (separated with mother or father or both) associated with self-harm behaviors? and (4) are there certain childhood separation characteristics (age at first parental leave, duration of parental separation) which are correlated with an increased risk of self-injuries?

## Materials and Methods

### Participants

This study is part of the research project named “Adolescent Health and Risky behaviors in Anhui Province” and was carried out *via* using a three-stage random cluster-sampling approach in Anhui province, China. Three cities—Tongling, Tianchang, and Fuyang, which are, respectively, located in southern, middle, and northern of Anhui province, were randomly selected by using a lottery method in the first stage. At the second stage, we use the same way to select one public middle school and high school from each city randomly. At last, eight targeted classes were included in our research randomly for the third stage. A total of 5,760 participants aged from 12 to 18 years complete the questionnaire with a response rate at 95.5%. We exclude 86 participants who are born in a single-parent family during their childhood. Finally, the questionnaires with missing responses in correlated questions were also excluded from this research (*n* = 746), and ultimately 4,928 participants, including 2,446 boys and 2,482 girls, were recruited in this study with the valid response rate at approximately 86.9% (4,928/5,674). The students were aged 12–18 years, and the mean (±SD) age was 14.73 ± 1.96 years. There were no significance differences of demographic variables between included samples and excluded samples. The present study was approved by the Biomedicine Ethical Committee of Anhui Medical University (20110136) and written informed consent was obtained from all subjects, all schools, and either of the students’ parents.

### Measures

#### Parent-Child Separation

A self-designed questionnaire (see Supplementary File 1) was used to survey various dimensions of parental separation. According to previous studies, children who have been separated from parents for at least 6 months are classified as “left-behind children” (Luo et al., 2012; Xu et al., 2018). Therefore, we focus on Chinese rural adolescents that have experienced at least 6 months of separation from either father or mother leaving their children in the hometown in the care by their grandparents or other relatives in order to find employment in the city. First at all, we asked the question of “whether you have been separated from your parents during your childhood?” The response option was dichotomized as follows: 0 = no and 1 = yes. Further, we investigated the details regarding their parental separation. Separation status was classified into four groups: no separation as reference group, only paternal separation (only having been separated from father), only maternal separation (only having been separated from mother), both-parents separation (having the experience of being separated from father and mother at the same time) called “parental separation” group. The age at first separation from father and mother is categorized as 0, 1–3, 4–6, or more than 6 years, corresponding roughly to commonly accepted developmental stages: infancy and toddlerhood, preschool childhood and school age.

#### Self Harm

The term self-harm is described as a series of self-inflicted and intentional behaviors that cause physical and psychological harm (Skegg, 2005). While in this study, five types of self-harm are measured (see Supplementary File 3), including highly lethal self-harm; less lethal self-harm with visible tissue damage; self-harm without visible tissue damage; self-harmful behaviors with latent damage; and psychological self-harm. Highly lethal self-harm is referred to as hanging, shooting, jumping from a high place, poisoning, stabbing, electrocution, and drowning, similar to the previously defined suicidal behaviors. Whereas less lethal self-harm with visible tissue damage (cutting and burning); self-harm without visible tissue damage (self-hitting, pinching, binding, as well as choking) in our study, are consistent with the classification of non-suicidal SH, including NSSI. Additionally, we have introduced two novel categories of self-harm into our research—self harmful behaviors with latent damage, like overconsuming alcohol, smoking too much, overeating and staying up too late, and psychological self-harm, such as insulting as well as despising—which were less studied previously. A total of 39 items are used to measure these five types of self-harm and the response options are dichotomized as follows: 0 = no and 1 = yes. Detailed information about this questionnaire has been described in our previous study (Han et al., 2018).

#### Covariates

We control the potential impact of several demographic variables on self-harm behavior involving gender (female or male), grade (from grade 7–12), maternal education (primary school, secondary school or college), paternal education (primary school, secondary school or college), relationship with mother (good or poor), relationship with father (good or poor), self-perceived family status and number of friends (<3 or ≥3). Since severity of depression is showed to be associated with NSSI, we also control depression as covariates in this study (Esposito-Smythers et al., 2010; Xiao et al., 2019).

The level of depression was evaluated *via* Self-Rating Depression Scale (SDS) which is introduced by Zung (1965). This scale is composed of 20 items with the total scores ranging from 20 to 80. The Cronbach’s alpha value was 0.88.

#### Reliability and Validity of the Measurements

After assessing by three experts in this field, the questionnaire was then carefully revised based on their comments. In order to guarantee suitability of content and language for study population, the questionnaire was retested with 156 middle and high school students within 1-week interval, showing Kappa values ranged from 0.81 to 0.96. Further, the consistency of the test was examined ranging from 0.74 to 0.88.

### Statistical Analysis

Statistics were analyzed by using SPSS for Windows (version 19.0; SPSS Inc., Chicago, IL, United States). Descriptive statistics are reported for all related factors and the prevalence of each type of self-harm. Then, we perform univariate logistic regression analysis to examine whether the sociodemographic features and depression score are related to each type of self-harm. Also, the relationship between parent-child separation and self-harm is explored. We took several sorts of parental separation into account—occurrence of parental separation, age at first separation and separation status (e.g., no separation, only maternal separation, only paternal separation and both-parents separation), as well as duration of separation. In the multivariate logistic regression models, we use different parameters of parent-child separation mentioned above as independent variables, each type of self-harm as dependent variable and gender, grade, relationship with mother, relationship with father and depression scale scores as covariates which were significant in the univariate analysis. In this study, the level of statistical significance is defined as *p* < 0.05 using two-side tests.

## Results

### Descriptive Statistics

After excluding the uncompleted questionnaires, the valid response rate was 81.7% with a total figure of 4,928. The sample (*n* = 4,928) consists of 2,446 males and 2,482 females aged from 12 to 18 years with a mean of 14.73 and standard deviation of 1.96. The prevalence rates for five subtypes of self-harm—highly lethal self-harm, less lethal self-harm with visible tissue damage, self-harm without visible tissue damage, self-harmful behaviors with latent damage, and psychological self-harm—were 6.2%, 20.2%, 31.9%, 19.9%, 22.8%, respectively. Nearly 30% (1406) of the participants experience separation from mother and nearly 40% (1886) have the history of separating from their father. As for separation status, the percentage of students who have both been separated from mother and father at the same time is 25% or so.

As shown in Table 1, it also demonstrates the prevalence of age at first separation from mother or father and the duration of maternal or paternal separation. In the univariate logistic analysis, gender, grade, relationship with father, relationship with mother, as well as depression are associated with five types of self-harm (*p* < 0.05), while maternal education and paternal education were not (*p* > 0.05).

**TABLE 1:** Prevalence of self-harm by sample characteristics (*N* = 4,928).

Category	*N* (%)	Highly lethal self-harm (%)	Less lethal self-harm with visible tissue damage (%)	Self-harm without visible tissue damage (%)	Self-harmful behaviors with latency damage (%)	Psychological self-harm (%)	Total self-harm (%)
**Gender**							
Male	2,446 (49.6)	130 (5.3)	340 (13.9)	742 (30.3)	467 (19.1)	486 (19.9)	1,019 (41.7)
Female	2,482 (50.4)	174 (7.0)	653 (26.3)	828 (33.4)	516 (20.8)	639 (25.7)	1,216 (49.0)
**Grade**							
Grade 7	904 (18.3)	35 (3.9)	123 (13.6)	207 (22.9)	90 (10.0)	116 (12.8)	296 (32.7)
Grade 8	861 (17.5)	54 (6.3)	192 (22.3)	289 (33.6)	174 (20.2)	198 (23.0)	427 (49.6)
Grade 9	868 (17.6)	63 (7.3)	192 (22.1)	269 (31.0)	170 (19.6)	189 (21.8)	371 (42.7)
Grade 10	791 (16.1)	53 (6.7)	174 (22.0)	242 (30.6)	176 (22.3)	201 (25.4)	357 (45.1)
Grade 11	754 (15.3)	50 (6.6)	142 (18.8)	259 (34.4)	169 (22.4)	203 (26.9)	378 (50.1)
Grade 12	750 (15.2)	49 (6.5)	170 (22.7)	304 (40.5)	204 (27.2)	218 (29.1)	406 (54.1)
**Maternal education**							
Primary school	2,247 (45.6)	148 (6.6)	480 (21.4)	751 (33.4)	501 (22.3)	561 (25.0)	1,077 (47.9)
Secondary school	2,582 (52.4)	147 (5.7)	487 (18.9)	785 (30.4)	465 (18.0)	544 (21.1)	1,115 (43.2)
College	99 (2.0)	9 (9.1)	26 (26.3)	34 (34.3)	17 (17.2)	20 (20.2)	43 (43.4)
**Paternal education**							
Primary school	865 (17.6)	61 (7.1)	193 (22.3)	293 (33.9)	188 (21.7)	234 (27.1)	434 (50.2)
Secondary school	3,786 (76.8)	226 (6.0)	739 (19.5)	1,190 (31.4)	749 (19.8)	830 (21.9)	1,682 (44.4)
College	277 (5.6)	17 (6.1)	61 (22.0)	87 (31.4)	46 (16.6)	61 (22.0)	119 (43.0)
**Relationship with mother**							
Poor	1,127 (22.9)	103 (9.1)	275 (24.4)	396 (35.1)	291 (25.8)	325 (28.8)	581 (51.6)
Good	3,801 (77.1)	201 (5.3)	718 (18.9)	1,174 (30.9)	692 (18.2)	800 (21.0)	1,654 (43.5)
**Relationship with father**							
Poor	1,584 (32.1)	135 (8.5)	391 (24.7)	574 (36.2)	392 (24.7)	440 (27.8)	834 (52.7)
Good	3,344 (67.9)	169 (5.1)	602 (18.0)	996 (29.8)	591 (17.7)	685 (20.5)	1,401 (41.9)
**Self-perceived family status**							
Poor	637 (12.9)	43 (6.8)	143 (22.4)	241 (37.8)	148 (23.2)	186 (29.2)	322 (50.5)
Intermediate	3,795 (77.0)	226 (6.0)	754 (19.9)	1,186 (31.3)	735 (19.4)	829 (21.8)	1,696 (44.7)
Good	496 (10.1)	35 (7.1)	96 (19.4)	143 (28.8)	100 (20.2)	110 (22.2)	217 (43.8)
**Number of friends**							
<3	1,193 (24.2)	90 (7.5)	282 (23.6)	435 (36.5)	251 (21.0)	330 (27.7)	598 (50.1)
≥3	3,735 (75.8)	214 (5.7)	711 (19.0)	1,135 (30.4)	732 (19.6)	795 (21.3)	1,637 (43.8)
**Maternal separation**							
No	3,522 (71.5)	203 (5.8)	652 (18.5)	1,034 (29.4)	652 (18.5)	753 (21.4)	1,481 (42.0)
Yes	1,406 (28.5)	101 (7.2)	341 (24.3)	536 (38.1)	331 (23.5)	372 (26.5)	754 (53.6)
**Paternal separation**							
No	3,042 (61.7)	168 (5.5)	527 (17.3)	866 (28.5)	538 (17.7)	644 (21.2)	1,245 (40.9)
Yes	1,886 (38.3)	136 (7.2)	466 (24.7)	704 (37.3)	445 (23.6)	481 (25.5)	990 (52.5)
**Separation status**							
No	2,844 (57.7)	151 (5.3)	485 (17.1)	795 (28.0)	496 (17.4)	585 (20.6)	1,141 (40.1)
Mother only	198 (4.0)	17 (8.6)	42 (21.2)	71 (35.9)	42 (21.2)	59 (29.8)	104 (52.5)
Father only	678 (13.8)	52 (7.7)	167 (24.6)	239 (35.3)	156 (23.0)	168 (24.8)	340 (50.1)
Both	1,208 (24.5)	84 (7.0)	299 (24.8)	465 (38.5)	289 (23.9)	313 (25.9)	650 (53.8)
**Age at first maternal separation**							
No	3,522 (71.5)	203 (5.8)	652 (18.5)	1,034 (29.4)	652 (18.5)	753 (21.4)	1,481 (42.0)
0–3 years old	217 (4.4)	17 (7.8)	58 (26.7)	83 (38.2)	67 (30.9)	72 (33.2)	128 (59.0)
3–6 years old	441 (8.9)	30 (6.8)	104 (23.6)	162 (36.7)	87 (19.7)	100 (22.7)	221 (50.1)
More than 6 years old	748 (15.2)	54 (7.2)	179 (23.9)	291 (38.9)	177 (23.7)	200 (26.7)	405 (54.1)
**Age at first paternal separation**							
No	3,042 (61.7)	168 (5.5)	527 (17.3)	866 (28.5)	538 (17.7)	644 (21.2)	1,245 (40.9)
0–3 years old	386 (7.8)	32 (8.3)	105 (27.2)	155 (40.2)	109 (28.2)	123 (31.9)	221 (57.3)
3–6 years old	576 (11.7)	42 (7.3)	145 (25.2)	225 (39.1)	131 (22.7)	141 (24.5)	313 (54.3)
More than 6 years old	924 (18.8)	62 (6.7)	216 (23.4)	324 (35.1)	205 (22.2)	217 (23.5)	456 (49.4)

### Parental Separation and Self-Harm

We examined the interactions between parental separation and the relationship with the parents and found no significant statistical interaction, suggesting that the effect of separation did not vary with such factor. Thus, we consider the main effect of parental separation. Table 2 shows that participants experienced maternal separation have a moderate relationship with four less lethal subtypes of self-harm (OR ranging from 1.32 to 1.48), while paternal separation was relevant to each type of self-harm (OR ranging from 1.28 to 1.57). Table 3 shows the odds ratios (ORs) and 95% confidence intervals (CIs) from the multivariable logistic regression analyses adjusted for gender, grade, and relationship with mother, relationship with father and depression. We find that besides the sorts of highly lethal self-harm, another four types of SH are still associated with maternal separation with odds ratio ranging from 1.29 to 1.49. By contrast, there are no differences in paternal separation showing five types of SH are linked with this group. When it comes to total self-harm, the results (see Table 3) show that it is correlated with both maternal and paternal separation after adjusting for several covariates.

**TABLE 2:** Unadjusted OR (95% CI) for self-harm by univariate analysis (*N* = 4,928).

Variables			Highly lethal self-harm	Less lethal self-harm with visible tissue damage	Self-harm without visible tissue damage	Self-harmful behaviors with latency damage	Psychological self-harm	Total self-harm
Gender	Female	Ref:						
	Male		0.74 (0.59–0.94)*	0.45 (0.39–0.52)*	0.87 (0.77–0.98)*	0.90 (0.78–1.03)	0.72 (0.63–0.82)*	0.74 (0.66–0.83)*
Grade	Grade 7	Ref:						
	Grade 8		1.66 (1.07–2.57)*	1.82 (1.42–2.34)*	1.70 (1.38–2.10)*	2.29 (1.74–3.01)*	2.03 (1.58–2.61)*	2.02 (1.67–2.45)*
	Grade 9		1.94 (1.27–2.97)*	1.80 (1.41–2.31)*	1.51 (1.22–1.87)*	2.20 (1.67–2.90)*	1.89 (1.47–2.44)*	1.53 (1.26–1.86)*
	Grade 10		1.78 (1.15–2.76)*	1.79 (1.39–2.31)*	1.48 (1.20–1.84)*	2.59 (1.97–3.41)*	2.31 (1.80–2.98)*	1.69 (1.39–2.06)*
	Grade 11		1.76 (1.13–2.75)*	1.47 (1.13–1.92)*	1.76 (1.42–2.19)*	2.61 (1.98–3.45)*	2.50 (1.94–3.22)*	2.07 (1.69–2.52)*
	Grade 12		1.74 (1.11–2.71)*	1.86 (1.44–2.40)*	2.30 (1.86–2.84)*	3.38 (2.58–4.43)*	2.78 (2.17–3.58)*	2.42 (1.99–2.96)*
Maternal education	Primary school	Ref:						
	Secondary school		0.86 (0.68–1.08)	0.86 (0.74–0.99)*	0.87 (0.77–0.98)*	0.77 (0.67–0.88)*	0.80 (0.70–0.92)*	0.83 (0.74–0.93)*
	College		1.42 (0.70–2.87)	1.31 (0.83–2.08)	1.04 (0.68–1.59)	0.72 (0.43–1.23)	0.76 (0.46–1.25)	0.83 (0.56–1.25)
Paternal education	Primary school	Ref:						
	Secondary school		0.84 (0.62–1.12)	0.84 (0.71–1.01)	0.89 (0.77–1.05)	0.89 (0.74–1.06)	0.76 (0.64–0.90)*	0.80 (0.69–0.92)*
	College		0.86 (0.50–1.50)	0.98 (0.71–1.36)	0.89 (0.67–1.20)	0.72 (0.50–1.02)	0.76 (0.55–1.05)	0.75 (0.57–0.98)*
Relationship with mother	Poor	Ref:						
	Good		0.56 (0.43–0.71)*	0.72 (0.62–0.85)*	0.83 (0.72–0.95)*	0.64 (0.55–0.75)*	0.66 (0.57–0.77)*	0.73 (0.63–0.83)*
Relationship with father	Poor	Ref:						
	Good		0.57 (0.45–0.72)*	0.67 (0.58–0.77)*	0.75 (0.66–0.85)*	0.65 (0.57–0.75)*	0.67 (0.58–0.77)*	0.65 (0.58–0.73)*
Self-perceived family status	Poor	Ref:						
	Intermediate		0.88 (0.62–1.23)	0.86 (0.70–1.05)	0.75 (0.63–0.89)*	0.79 (0.65–0.97)*	0.68 (0.56–0.82)*	0.79 (0.67–0.94)*
	Good		1.05 (0.66–1.67)	0.83 (0.62–1.11)	0.67 (0.52–0.86)*	0.83 (0.63–1.11)	0.69 (0.53–0.91)*	0.76 (0.60–0.96)*
Depression			1.11 (1.09–1.13)*	1.06 (1.05–1.08)*	1.05 (1.04–1.06)*	1.07 (1.06–1.08)*	1.07 (1.06–1.09)*	1.05 (1.04–1.06)*
Maternal separation	No	Ref:						
	Yes		1.27 (0.99–1.62)	1.41 (1.22–1.64)*	1.48 (1.30–1.69)*	1.36 (1.17–1.57)*	1.32 (1.15–1.53)*	1.59 (1.41–1.81)*
Paternal separation	No Yes	Ref:	1.33 (1.05–1.68)*	1.57 (1.36–1.80)*	1.50 (1.33–1.69)*	1.44 (1.25–1.66)*	1.28 (1.11–1.46)*	1.60 (1.42–1.79)*
Separation status	No	Ref:						
	Mother only		1.68 (0.99–2.83)	1.31 (0.92–1.87)	1.44 (1.07–1.95)*	1.27 (0.89–1.82)	1.64 (1.19–2.25)*	1.65 (1.24–2.20)*
	Father only		1.48 (1.07–2.05)*	1.59 (1.30–1.94)*	1.40 (1.18–1.68)*	1.42 (1.15–1.73)*	1.27 (1.05–1.55)*	1.50 (1.27–1.78)*
	Both		1.33 (1.01–1.76)*	1.60 (1.36–1.88)*	1.61 (1.40–1.86)*	1.49 (1.26–1.75)*	1.35 (1.15–1.58)*	1.74 (1.52–1.99)*
Age at first maternal separation (years old)	No	Ref:						
	0–3		1.39 (0.83–2.33)	1.61 (1.18–2.20)*	1.49 (1.12–1.98)*	1.97 (1.46–2.66)*	1.83 (1.36–2.45)*	1.98 (1.50–2.62)*
	3–6		1.19 (0.80–1.78)	1.36 (1.07–1.72)*	1.40 (1.14–1.72)*	1.08 (0.84–1.39)	1.08 (0.85–1.37)	1.38 (1.14–1.69)*
	>6		1.27 (0.93–1.74)	1.39 (1.15–1.67)*	1.53 (1.30–1.80)*	1.36 (1.13–1.65)*	1.34 (1.12–1.61)*	1.63 (1.39–1.91)*
Age at first paternal separation (years old)	No	Ref:						
	0–3		1.55 (1.04–2.29)*	1.78 (1.40–2.27)*	1.69 (1.36–2.10)*	1.83 (1.44–2.33)*	1.74 (1.38–2.19)*	1.93 (1.56–2.40)*
	3–6		1.35 (0.95–1.91)	1.61 (1.30–1.98)*	1.61 (1.34–1.94)*	1.37 (1.10–1.70)*	1.21 (0.98–1.49)	1.72 (1.44–2.06)*
	>6		1.23 (0.91–1.66)	1.46 (1.22–1.74)*	1.36 (1.16–1.59)*	1.33 (1.11–1.59)*	1.14 (0.96–1.36)	1.41 (1.21–1.63)*
Maternal separation duration			1.03 (0.99–1.08)	1.06 (1.03–1.09)*	1.06 (1.03–1.08)*	1.03 (1.00–1.06)*	1.03 (1.00–1.06)*	1.07 (1.04–1.09)*
Paternal separation duration			1.04 (0.99–1.08)	1.07 (1.04–1.09)*	1.07 (1.05–1.09)*	1.07 (1.04–1.09)*	1.04 (1.02–1.07)*	1.07 (1.05–1.10)*

*p < 0.05.

**TABLE 3:** Multivariable logistic regression analysis showing the AOR (95% CI) between parent-child separation and five subtypes of self-harm.

Variables			Highly lethal self-harm *^a^*	Less lethal self-harm with visible tissue damage *^a^*	Self-harm without visible tissue damage *^a^*	Self-harmful behaviors with latency damage *^a^*	Psychological self-harm *^a^*	Total self-harm *^a^*
Maternal separation	No	Ref:						
	Yes		1.20 (0.93–1.55)	1.41 (1.21–1.65)*	1.49 (1.31–1.71)*	1.33 (1.14–1.56)*	1.29 (1.11–1.50)*	1.59 (1.40–1.81)*
Paternal separation	No	Ref:						
	Yes		1.29 (1.01–1.64)*	1.55 (1.34–1.80)*	1.50 (1.32–1.70)*	1.41 (1.22–1.64)*	1.22 (1.06–1.41)*	1.58 (1.40–1.79)*
Separation status	No	Ref:						
	Mother only		1.60 (0.93–2.75)	1.38 (0.96–2.00)	1.41 (1.04–1.92)*	1.21 (0.85–1.75)	1.64 (1.18–2.28)*	1.63 (1.21–2.20)*
	Father only		1.48 (1.06–2.08)*	1.60 (1.29–1.97)*	1.39 (1.16–1.67)*	1.38 (1.11–1.70)*	1.21 (1.00–1.49)*	1.47 (1.24–1.76)*
	Both		1.26 (0.95–1.68)	1.59 (1.34–1.88)*	1.63 (1.41–1.88)*	1.46 (1.23–1.74)*	1.30 (1.10–1.53)*	1.74 (1.51–2.00)*
Age at first maternal separation (years old)	No	Ref:						
	0–3		1.30 (0.77–2.20)	1.56 (1.13–2.17)*	1.48 (1.11–1.98)*	1.95 (1.43–2.66)*	1.80 (1.32–2.44)*	1.96 (1.47–2.61)*
	3–6		1.15 (0.77–1.73)	1.39 (1.08–1.77)*	1.45 (1.17–1.80)*	1.10 (0.85–1.43)	1.09 (0.85–1.39)	1.43 (1.16–1.75)*
	>6		1.20 (0.87–1.66)	1.38 (1.13–1.68)*	1.52 (1.28–1.80)*	1.31 (1.08–1.59)*	1.28 (1.06–1.55)*	1.60 (1.36–1.89)*
Age at first paternal separation (years old)	No	Ref:						
	0–3		1.47 (0.98–2.20)	1.72 (1.33–2.21)*	1.69 (1.36–2.12)*	1.84 (1.43–2.36)*	1.69 (1.33–2.15)*	1.89 (1.52–2.36)*
	3–6		1.32 (0.92–1.89)	1.64 (1.32–2.04)*	1.66 (1.37–2.01)*	1.39 (1.11–1.73)*	1.20 (0.96–1.49)	1.77 (1.47–2.13)*
	>6		1.19 (0.87–1.63)	1.44 (1.19–1.73)*	1.34 (1.14–1.57)*	1.27 (1.05–1.53)*	1.07 (0.89–1.28)	1.37 (1.18–1.60)*
Duration of maternal separation			1.02 (0.97–1.07)	1.06 (1.03–1.09)*	1.05 (1.03–1.08)*	1.02 (1.00–1.06)*	1.02 (1.00–1.06)*	1.06 (1.04–1.09)*
Duration of paternal separation			1.03 (0.99–1.07)	1.06 (1.04–1.09)*	1.06 (1.04–1.09)*	1.06 (1.03–1.08)*	1.03 (1.01–1.06)*	1.07 (1.04–1.09)*

*^a^Adjusted for gender, grade, relationship with mother, relationship with father and depression scale scores.*

*p < 0.05.

### Separation Status and Self-Harm

When it comes to the separation status, paternal separation is associated with each type of self-harm compared to no separation group, whereas maternal separation is solely linked with self-harm without visible tissue damage (OR:1.44, 95%CI:1.07–1.95, *P* < 0.05) and psychological self-harm (OR:1.64, 95%CI:1.19–2.25, *P* < 0.05) when compared to control group. After adjusted for covariates, the relation seems alike. It shows paternal separation is still related to five types of SH and maternal separation is significantly relative to self-harm without visible tissue damage and psychological SH with adjusted odds ratio ranging from 1.41 to 1.64. In addition, each type of separation status (only maternal separation, only paternal separation and both-parent separation) is related to total self-harm with ORs ranging from 1.47 to 1.74 (see Table 3).

### Duration of Separation and Self-Harm

As shown in Table 2, except for highly lethal self-harm, the other four less lethal SH subtypes demonstrate a relation with both lengths of paternal separation and maternal separation. Additionally, when adjusted by covariates which are significant in the univariate analysis, it shows similar relationships, with the adjusted odds ratio ranging from 1.02 to 1.06 (see Table 3).

### Age at First Separation and Self-Harm

Considering different stages of childhood life, we divide participants into four groups: no separation group; separation groups with the age for first departure at 0–3 years old, 4–6 years old and more than 6 years old separately. In Table 2, we found that two sorts of SH—less lethal self-harm with visible tissue damage and self-harm without visible tissue damage—are associated with variation for age at first separation. Nevertheless, from the results of multivariable logistic regression analysis, it can be seen that these two groups are also linked to the age of initial separation and additional self-harmful behaviors with latent damage group showed an association with paternal separation. Furthermore, separation from parents at each subgroup is associated with total self-harm (see Table 3). We also performed a multivariate logistic regression analysis to confirm the relationships between parental separation and each SH subtype, with adjustments for all sociodemographic variables and depression. The results showed similar relationships (see Supplementary File 2: Supplementary Table 1).

## Discussion

In the United States, paternal separation is common due to divorce. In parts of Mexico, paternal separation is common due to migrant working status. Whereas in China, a growing number of children in rural areas have experienced separation from their parents in recent decades due to migration for better work. Self-harm behavior is a serious problem in the contemporary society, which deserves our seriously attention. Hence, it is urgent to further understand the psychological adjustment of these children. Considering very limited studies published have addressed the SH outcomes of left-behind children in China, we examine the association between parent-child separation and five subtypes of self-harm behaviors in large numbers of sample adolescents in Anhui province. Consistent with previous studies, our results indicate that separation from their parents, especially paternal separation, in childhood other than parental death increase the risks for future self-harm prevalence in adolescence. Results from this research may provide a novel insight into the phenomenon of parent-child separation and into its effect in adolescents’ self-harm.

### Parental Separation and Self-Harm

In the univariate analysis, paternal separation is associated with each type of SH, while maternal separation is associated with four types of SH among Chinese adolescents, but not linked with highly lethal self-harm. Similarly, these relationships still exist after adjusting for a cluster of covariates. Further, unlike maternal separation, paternal separation was significantly associated with each SH subtype, supporting the significant role of paternal separation. These findings serve as a reminder that paternal separation may have a more robust relationship with SH than maternal separation in our study. Contradictorily, the current study found that teenagers without mother’ company represent a higher risk for attempted suicide, in contrast to those without father’s company (Knipe et al., 2019). As for less lethal self-harm with visible tissue damage, self-harm without visible tissue damage, the findings were consistent with research that has repeatedly identified associations between parental separation and NSSI (Lan et al., 2019). As shown in our study, having been separated from both parents reflected a connection with less lethal self-harm. There is evidence that absence of both parents may have a negative impact on children’s development and mental or physical health (Chang et al., 2017). Based on this finding, we can deduce that strategies toward the prevention of self-harm in adolescents need to pay more attention to individuals who have experienced separation, especially both separation from parents. Nowadays, more and more parents in rural areas would choose to migrate to urban areas for better employment and leave their children behind at home living with grandparents. On the one hand, in spite of grandparents’ willingness to childcare, they are actually incapable of undertaking the accountability in keeping children from risks to self-harm. The significant generation gap between them would probably hinder their effective communication and emotional linkages, and finally would lead to later well-being distress. Furthermore, previous studies indicated that grandparents raising was associated with an increased risk of depression (Minkler et al., 1997; Hadfield, 2014). Another study reveals that the absence of parents during childhood may influence children’s mood, which is consistent with attachment theory (Lai and Carr, 2018; Zhao et al., 2018). Parents migrate to urban areas would not have enough time and energy to care for their children, those who were left behind usually experience less parent care and communication, which would have a detrimental effect to emotional well-being (Zhao et al., 2018) and increase the likelihood to adolescent self-harm. Indeed, parental separation would contribute to a child’s problems of well-being owing to the emotional impacts as well as inadequate care and support from their parents, which we speculate could lead to further self-harm behaviors. The mechanisms underlying parental separation are complex. Future research still needs to explore the details underlying parent-child separation from parents.

### Characteristics of Parental Separation and Self-Harm

There are two forms of separation from their parents—age at first separation and duration of parental separation. To date, few studies have adequately explored the relationship of two formats of separation and sub-types of self-harm. In our study, our aim is to find a link between SH and separation and provide some potentially credible ways to prevent self-harm in adolescents.

The relationship between parental separation and highly lethal self-harm identified is distinct from the relationships with the non-lethal SH subtypes in our study. Our research suggests age at first separation is correlated with four types non-lethal SH subtypes but have no association with highly lethal self-harm. This finding may indicate that parent-child separation would not be the risk factor for highly lethal self-harm. According to previous study, non-lethal SH subtypes differ from highly lethal self-harm by nature as genetics may cause vulnerability to suicidal attempts among adolescents (Mirkovic et al., 2016). Furthermore, our study shows that individuals who suffer paternal separation prior to age of three are at most risk with the highest OR, suggesting participants who suffer from paternal migration when they are less than 3 years may be more prone to future self-harm intentions and behaviors. Notably, the result also indicates association between paternal separation before 3 years and psychological self-harm, which further confirms that age between 0–3 at separation is more susceptible to future self-harm in adolescent. When it comes to less lethal self-harm with visible tissue damage and self-harm without visible tissue damage, we can conclude that parental separation at each sub-group (0–3 years old, 4–6 years old and more than 6 years old) would increase the risk for self-harm, suggesting a lack of parental company in essential period relates to an increased risk for self-harm. In brief, our results would support the classification of self-harm into five subtypes so as to figure out potential risk factors for the occurrence of self-harm, which would help to discover novel interventions for adolescent self-harm more precisely. Alternatively, we find that the duration of maternal separation and paternal separation are predictive of four types of self-harm except for highly lethal self-harm. The longer they are exposed to paternal separation, the more prone they are to self-harm. The results are consistent with the previous study, which indicates that the duration of parent-child separation is related to psychological adjustment and that longer separation would ultimately lead to poorer adjustment (Xu et al., 2018). All in all, these results provide a novel insight that those adolescents suffering lengthy separation are at higher risk to develop self-harm who deserves our attention. However, these findings are best considered as preliminary data which require further validation.

The accumulated evidence shows that early separation is a risk factor for their self-harm tendency. The relatively high risks of paternal separation may shed more light on the importance of early parenting. This results may be consistent with previous research which underscores the significance of early parenting (Liu et al., 2009). Surprisingly, it is unveiled that father involvement may exert a stronger effect on behavior and emotion of adolescents in a longitudinal study (Flouri and Buchanan, 2003). It means that father plays a critical role in emotional comprehension (Psychogiou et al., 2018). Therefore, we speculate that absence of father may result in adolescent emotional disorder, which may evoke self-injury. While considering age at first separation and duration of separation from mother, this trend may not be that pronounced. Future research still needs to find the exact trend underlying these phenomena.

Our major findings are of vital significance to prevent adolescents from self-harming. First and foremost, the striking prevalence in general adolescents highlights the importance of self-harm interventions to decrease the prevalence of these behaviors. Besides, our study also stresses the critical role of a father’s company and this may provide a novel intervention direction for preventing self-harm.

The limitations of this study should be noted. First, limited by the nature of this study, we cannot draw a conclusion to reveal the causality between parent-child separation and self-harm behavior among adolescents. Further studies should be conducted by using a prospective design in order to elucidate cause and effect relationship between them. Secondly, only students in three different locations of Anhui province are selected; it cannot be the representatives of overall Chinese adolescents. Thirdly, there exists a recalling bias in this cross-sectional study. the statistics of age at initial separation and duration of separation cannot be memorized clearly, the Since data are not exactly accurate on this account. Lastly, the data are collected from three middle schools and we do not take those who dropped out of school with poor performance or in poverty into account. These people are more susceptible to self-harm behaviors. The association between parental leave and SH is likely to be overstated or understated. Future research should take this part of teenagers on board and recruit participants from all manners of sources.

## Conclusion

Despite those limitations delineated above, we believe that our study provides critical insights into the association between parent-child separation and self-harm. Actually, this research has aroused our awareness of long-term negative effects of parent-child separation and proposed a novel strategy to decrease SH prevalence among adolescents. Nevertheless, the mechanism underlying this relationship remains unclear. Further studies should be conducted to disclose it.

## Data Availability Statement

The original contributions presented in the study are included in the article/Supplementary Material, further inquiries can be directed to the corresponding author/s.

## Ethics Statement

The studies involving human participants were reviewed and approved by Biomedicine Ethical Committee of Anhui Medical University. Written informed consent to participate in this study was provided by the participants’ legal guardian/next of kin.

## Author Contributions

P-YS conceived and designed the study. T-JZ and G-FW were involved in the collection of the data and performed the statistical analysis. T-JZ and H-YR drafted the article. G-DX and M-YY contributed to the collection of the data. T-JZ and M-YY revised the article. All authors gave their comments on the article and approved the final version.

## Conflict of Interest

The authors declare that the research was conducted in the absence of any commercial or financial relationships that could be construed as a potential conflict of interest.

## Publisher’s Note

All claims expressed in this article are solely those of the authors and do not necessarily represent those of their affiliated organizations, or those of the publisher, the editors and the reviewers. Any product that may be evaluated in this article, or claim that may be made by its manufacturer, is not guaranteed or endorsed by the publisher.
