# Supplementary material for: Childhood Separation From Parents and Self-Harm in Adolescence: A Cross-Sectional Study in Mainland China
Source: Front Psychol. 2022 Jan 26;12:645552. doi: 10.3389/fpsyg.2021.645552 (PMC8825502; doi:10.3389/fpsyg.2021.645552)
Supplement: Supplementary file 1 [file Data_Sheet_1.doc]

**Additional File 1:**

**Self-designed questionnaire about parental separation**

1. Whether you have been separated from your mother during your childhood? (have been separated: refers to not being able to live with your family and lasting at least 6 months at a time; near home, those who leave early and return late do not count)

[A] no [B] yes

(If you choose [A], please do question 4 directly; if you choose [B], do the following in order)

1. What is your age when your mother was separated from you for the first time (separated from you for at least 6 months)?
2. Less than one year old [B] one to three years old

[C] four to six years old [D] more than six years old

1. Before you started junior high school, your mother has worked abroad for ___ months.(work abroad: refer to migration to other city and not living with you)
2. Whether you have been separated from your father during your childhood? (have been separated: refers to not being able to live with your family and lasting at least 6 months at a time; near home, those who leave early and return late do not count)

[A] no [B] yes

(If you choose [A], then go into question 7; if you choose [B], do the following in order)

1. What is your age when your father was separated from you for the first time (separated from you for at least 6 months)?

[A] Less than one year old [B] one to three years old

[C] four to six years old [D] more than six years old

1. Before you started junior high school, your father has worked abroad for ___ months.(work abroad: refer to migration to other city and not living with you)
2. Whether you have been separated from your father and mother at the same time during your childhood? (have been separated: refers to not being able to live with your family and lasting at least 6 months at a time; near home, those who leave early and return late do not count)
3. no [B] yes

(If you choose [A], then finish this part; if you choose [B], do the following in order)

1. What is your age when your parents was separated from you for the first time (separated from you for at least 6 months with both parents)?

[A] Less than one year old [B] one to three years old

[C] four to six years old [D] more than six years old

1. Before you started junior high school, your father has worked abroad for ___ months.(work abroad: refer to migration to other city and not living with you; separated from you for at least 6 months with both parents)
