# Supplementary material for: Childhood Separation From Parents and Self-Harm in Adolescence: A Cross-Sectional Study in Mainland China
Source: Front Psychol. 2022 Jan 26;12:645552. doi: 10.3389/fpsyg.2021.645552 (PMC8825502; doi:10.3389/fpsyg.2021.645552)
Supplement: Supplementary file 2 [file Data_Sheet_2.doc]

**Additional File 2**

**TABLE S1** Multivariable logistic regression analysis showing the AOR (95% CI) between parent-child separation and five subtypes of self-harm(N=4928).

| **Variables** | | | **Highly lethal**  **self-harm ^a^** | **Less lethal self-harm with visible tissue damage ^a^** | **Self-harm without visible tissue damage ^a^** | **Self-harmful behaviors**  **with latency damage ^a^** | **Psychological**  **self-harm ^a^** | **Total self-harm ^a^** |
| --- | --- | --- | --- | --- | --- | --- | --- | --- |
| Maternal separation | No | Ref: |  |  |  |  |  |  |
|  | Yes |  | 1.23(0.95-1.59) | 1.42(1.22-1.67)* | 1.49(1.30-1.71)* | 1.34(1.15-1.57)* | 1.29(1.11-1.50)* | 1.60(1.40-1.82)* |
| Paternal separation | No | Ref: |  |  |  |  |  |  |
|  | Yes |  | 1.32(1.03-1.69)* | 1.58(1.36-1.84)* | 1.51(1.33-1.72)* | 1.42(1.22-1.65)* | 1.22(1.05-1.41)* | 1.59(1.41-1.80)* |
| Separation status | No | Ref: |  |  |  |  |  |  |
|  | Mother only |  | 1.67(0.97-2.87) | 1.41(0.97-2.04) | 1.40(1.03-1.91)* | 1.24(0.86-1.79) | 1.63(1.17-2.28)* | 1.65(1.22-2.22)* |
|  | Father only |  | 1.51(1.07-2.13)* | 1.62(1.31-2.00)* | 1.39(1.16-1.68)* | 1.38(1.11-1.71)* | 1.21(0.98-1.49) | 1.48(1.24-1.77)* |
|  | Both |  | 1.30(0.98-1.74) | 1.62(1.36-1.93)* | 1.64(1.41-1.90)* | 1.48(1.24-1.76)* | 1.30(1.10-1.53)* | 1.75(1.51-2.02)* |
| Age at first maternal separation (years old) | No | Ref: |  |  |  |  |  |  |
|  | 0-3 |  | 1.32(0.78-2.25) | 1.59(1.15-2.20)* | 1.49(1.11-1.99)* | 1.95(1.43-2.67)* | 1.79(1.32-2.43)* | 1.96(1.47-2.61)* |
|  | 3-6 |  | 1.18(0.78-1.78) | 1.40(1.09-1.80)* | 1.45(1.17-1.80)* | 1.11(0.86-1.44) | 1.07(0.84-1.38) | 1.43(1.16-1.76)* |
|  | > 6 |  | 1.23(0.89-1.70) | 1.39(1.14-1.69)* | 1.52(1.28-1.80)* | 1.32(1.08-1.60)* | 1.28(1.06-1.55)* | 1.60(1.36-1.89)* |
| Age at first paternal separation (years old) | No | Ref: |  |  |  |  |  |  |
|  | 0-3 |  | 1.49(0.99-2.25) | 1.74(1.35-2.25)* | 1.71(1.36-2.14)* | 1.84(1.43-2.36)* | 1.67(1.31-2.14)* | 1.89(1.51-2.36)* |
|  | 3-6 |  | 1.37(0.95-1.97) | 1.69(1.35-2.11)* | 1.68(1.38-2.03)* | 1.40(1.11-1.75)* | 1.20(0.97-1.50) | 1.79(1.49-2.16)* |
|  | > 6 |  | 1.22(0.89-1.67) | 1.46(1.20-1.76)* | 1.34(1.14-1.58)* | 1.28(1.06-1.55)* | 1.06(0.88-1.28) | 1.37(1.18-1.61)* |
| Duration of maternal separation |  |  | 1.02(0.97-1.07) | 1.06(1.03-1.09)* | 1.05(1.03-1.08)* | 1.02(1.00-1.06)* | 1.02(1.00-1.05)* | 1.06(1.04-1.09)* |
| Duration of paternal separation |  |  | 1.03(0.99-1.07) | 1.06(1.04-1.09)* | 1.06(1.04-1.09)* | 1.06(1.03-1.09)* | 1.03(1.00-1.05)* | 1.07(1.04-1.09)* |

^a^ Adjusted for gender, grade, maternal education, paternal education, relationship with mother, relationship with father, self-perceived family status, numbers of friends, and depression scale scores.

* *p* < 0.05
