# Supplementary material for: Childhood Separation From Parents and Self-Harm in Adolescence: A Cross-Sectional Study in Mainland China
Source: Front Psychol. 2022 Jan 26;12:645552. doi: 10.3389/fpsyg.2021.645552 (PMC8825502; doi:10.3389/fpsyg.2021.645552)
Supplement: Supplementary file 3 [file Data_Sheet_3.doc]

**Highly lethal self-harm**

1. hanging;
2. jumping from a high place;
3. poisoning (e.g., herbicide, pesticide, or carbon monoxide);
4. cutting blood vessels deliberately (e.g., cutting wrist, neck, or fatal parts of the blood vessels);
5. stabbing;
6. electrocution;
7. drowning;
8. overdosing (e.g., hypnagogues);
9. recreational drug ingestion

**Less lethal self-harm with visible tissue damage**

1. cutting (e.g., arms, legs, or other parts except for fatal parts of the body);
2. burning;
3. self-biting;
4. scratching;
5. gouging;
6. carving words or symbols into skin;
7. sticking needles or pins into skin;
8. interfering with wound healing.

**Self-harm without visible tissue damage**

1. selfhitting;
2. banging head or fist against something;
3. deliberatefrostbite;
4. pinching;
5. malaxating;
6. binding;
7. pulling hair;
8. choking.

**Self-harmful behaviours with latency damage**

1. exercising to hurt oneself;
2. denying oneself a necessity as punishment;
3. stopping medication or starving with intent to cause harm;
4. deliberate recklessness (e.g., risk-taking with cars or trains);
5. having intercourse with another (not for the purpose of money or love);
6. overconsuming alcohol (e.g., alcoholism or drinking beyond one’s endurance capacity);
7. smoking too much;
8. overeating;
9. staying up too late (not for working, learning, or entertainment).

**Psychological self-harm**

1. closing oneself off (forcing oneself to reduce or cease contact with the outside word);
2. distancing oneself from friends on purpose;
3. making oneself unpopular among friends on purpose;
4. insulting oneself;
5. despising oneself.
